# Bones in the Wrong Place: A Case of Palpable Penile Vascular Calcification as a Urologic Manifestation of Uncontrolled Diabetes

**DOI:** 10.51894/001c.144654

**Published:** 2025-09-30

**Authors:** Seth Thomas

**Affiliations:** 1 Department of Urology U of M Health Sparrow

## 128

We present the case of a young, male patient, suffering from a several genitourinary disease processes secondary to uncontrolled diabetes, including completely calcified distal branches of the internal pudendal artery within the penile shaft, which is rarely reported in the literature, and were visible on CT scan and palpable on physical examination. A 34-year-old male presented to the emergency department with a chief complaint of abdominal pain. At the time of presentation, a CT scan of the abdomen and pelvis was performed, demonstrating a distended bladder and bilateral, symmetric, hyper-attenuating structures within the penis, medial to the corpora cavernosa. The urology service was consulted for urethral foreign body, however, a foley catheter was placed without difficulty and the structures had been present 4 years prior on CT scan. On physical exam, a bone-like structure could be palpated within the penile shaft, adjacent to the foley catheter, preventing bending of the penis. Many urologic conditions are known to be caused by, or at least worsened by uncontrolled diabetes. We present the case of a young male patient with neurogenic bladder, erectile dysfunction, and a rarely reported finding in the literature of complete calcification of distal branches of the internal pudendal artery, limiting traditional erectile dysfunction treatment options.

